# STAT1 modification improves therapeutic effects of interferons on lung cancer cells

**DOI:** 10.1186/s12967-015-0656-0

**Published:** 2015-09-08

**Authors:** Junjie Chen, Jialu Zhao, Lefu Chen, Nian Dong, Zhaojian Ying, Zhenzhen Cai, Dongxiang Ji, Yong Zhang, Li Dong, Yuping Li, Lei Jiang, Michael J. Holtzman, Chengshui Chen

**Affiliations:** Department of Respiratory and Critical Care Medicine, The First Affiliated Hospital of Wenzhou Medical University, Wenzhou, 325000 Zhejiang China; Department of Pulmonary Medicine, Lishui Central Hospital, The Fifth Affiliated Hospital of Wenzhou Medical University, Lishui Hospital of Zhejiang University, Lishui, Zhejiang China; Department of Neurosurgery, The First Affiliated Hospital of Wenzhou Medical University, Wenzhou, Zhejiang China; Drug Discovery Program, Pulmonary and Critical Care Medicine, Department of Medicine, Washington University, St. Louis, MO USA; Central Laboratory, The First Affiliated Hospital of Wenzhou Medical University, Wenzhou, Zhejiang China

**Keywords:** Signal transducer and activator of transcription 1, Interferons, Lung cancer, Cell proliferation, Migration

## Abstract

**Background:**

Interferons (IFNs) have potent anti-proliferative, pro-apoptotic, and immunomodulatory activities against cancer. However, the clinical utility of IFNs is limited by toxicity and pharmacokinetics making it difficult to achieve sustained therapeutic levels especially in solid tumors.

**Methods:**

Signal Transducer and Activator of Transcription 1 (STAT1) or a modified STAT1 (designated STAT1-CC) that is hyper-responsive to IFN were overexpressed in lung cancer SPC-A-1 and H1299 cells using lentiviral vectors. Transduction efficiency was monitored using enhanced green fluorescent protein (EGFP) expression. After transduction, cells were treated with interferon-gamma (IFN-γ) or interferon-beta (IFN-β) and monitored for cell proliferation, migration, and invasiveness using Cell Counting Kit-8 and transwell chamber assays and for apoptosis using Annexin V detection by flow cytometry. In addition, levels of STAT1, STAT1 Tyr-701 phosphorylation (pSTAT1), fibronectin, and β-catenin were determined using western blotting. In the case of IFN-γ stimulation, levels of S100A4, proliferating cell nuclear antigen (PCNA), and c-fos expression were also determined.

**Results:**

We found that expression of STAT1 or STAT1-CC enhanced the effect of IFN-γ and, IFN-β on inhibition of human lung cancer cell proliferation, migration and invasiveness. Moreover, STAT1 and STAT1-CC expression caused increases in pSTAT1 and decreases in fibronectin and β-catenin levels. STAT1-CC showed increased effects compared to STAT1 on IFN-γ induced pSTAT1 and down-regulation of S100A4, PCNA, and c-fos levels.

**Conclusion:**

The results show that STAT1-CC exhibited more strength in improving the antitumor response of IFNs in lung cancer cells. Results from this study suggest that combined treatment of IFNs and STAT1-CC might be a feasible approach for the clinical management of lung cancer in the future.

## Background

Lung cancer is a highly malignant disease with a dismal prognosis. For example, 80–85 % of lung cancer cases are categorized as non-small cell lung cancers [1], and the 5-year overall survival rate of non-small cell lung cancer cases remains at less than 10 % despite improvements in surgery, radiotherapy, and chemotherapy [2]. Thus, novel treatment strategies are needed to improve the outcomes of lung cancer patients.

The use of interferons (IFNs) could be a potential strategy in the treatment of lung cancer [3]. IFNs have been shown to have anti-proliferative, anti-angiogenic, pro-apoptotic, and immunoregulatory effects [3]. Type I IFNs (the IFN-α family and IFN-β) have been used with some success for the treatment of several types of cancer, including hematological malignancies and solid tumors [4]. Type II, IFN-γ, also has antitumor activities in various types of cancer [5, 6]. Unfortunately, although IFNs have impressive potential activities against cancer, the clinical utility of IFNs in the treatment of solid tumors has been limited [3, 5]. The half-life of the intra-tumor concentration was short, thus requiring high doses of IFNs for a therapeutic effect that would result in toxic side effects [3, 5].

The IFN/STAT1 pathway is a typical signaling pathway that mediates crosstalk between tumor cells and components of the host microenvironment [7, 8]. IFN activities rely on signaling through three types of IFN receptors (for type I, II, and III IFNs) and the Janus activated kinase-signal transducer and activator of transcription (JAK-STAT) pathway that includes receptor-associated JAKs and STATs as well as downstream modulators, transcription factors, enhancers, and coactivators. STAT1 is a central mediator for IFN-related intracellular signaling and is considered a tumor suppressor protein related to the IFNs [9]. The role of STAT1 in the IFN-signaling pathway in the context of its antiviral actions has been extensively studied [10]. Previous studies have shown that modified STAT1 (STAT1-CC, the SH2 domain of STAT1 Ala-656 and Asn-658 site with double-cysteine alternative) broadly improves IFN signal transduction in human cells [11]. Compared to wild-type STAT1, STAT1-CC increases the capacity of STAT1 Tyr-701 phosphorylation (pSTAT1), nuclear translocation, DNA binding, recruitment of p300/CBP coactivator, and thus IFN efficacy. Encephalomyocarditis viral replication is inhibited in STAT1-CC-expressing cells, a finding which further extends the benefits of STAT1-CC [11]. Overexpression of STAT1-CC in a IFN-resistant cell line results in IFN-inducible control of viral replication and viral clearance, and increases the anti-viral effect in liver cells that are resistant to IFN as well [12].

It is still unclear whether using the gain-of-function of STAT1-CC could significantly improve the anti-tumor response of IFNs in lung cancer. To address this issue, our present study examined the outcome with overexpression of STAT1 or STAT1-CC genes in lung cancer cells. Since fibronectin, β-catenin, S100A4, proliferating cell nuclear antigen (PCNA) and c-fos have been reported to be involved in the control of cell growth or metastasis in cancers, we also evaluated the effect of STAT1-CC on the expression of these proteins in lung cancer cells. The results demonstrate that STAT1-CC has strong anti-tumor effects in lung cancer cells in response to IFNs and indicate that combined treatment of IFNs and STAT1-CC could be of potential value for further research in vivo and in an eventual therapeutic approach for treatment of human lung cancer.

## Methods

### Cell culture

Human lung cancer cell lines SPC-A-1 and H1299, and human embryonic kidney 293T cells were obtained from the Type Culture Collection of the Chinese Academy of Sciences, Shanghai, China. SPC-A-1 and H1299 cell lines were maintained in RPMI 1640 supplemented with 10 % fetal bovine serum (FBS) and 100 U/ml penicillin/streptomycin (Invitrogen, Carlsbad, CA). Human embryonic kidney 293T cells were maintained in Dulbecco’s modified Eagle’s medium supplemented with 10 % FBS and 100 U/ml penicillin/streptomycin. Cells were cultured in a humidified 37 °C incubator with 5 % CO_2_.

### Lentiviral vector construction and transduction

**Fig. 1:**
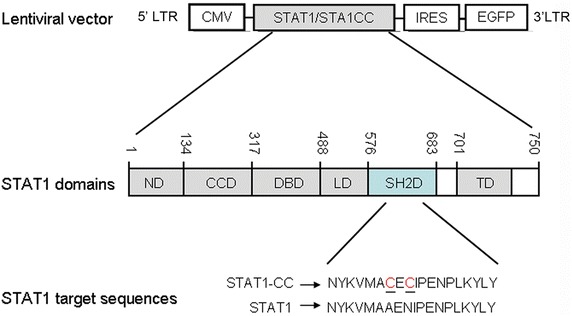
Lentiviral vector constructs for STAT1 and STAT1-CC. Diagram illustrates the target sequence of the sites for double-cysteine mutations in the SH2 domain of STAT1 and subsequent orientation into STAT1 and then into lentiviral vectors expressing EGFP with intervening IRES. The abbreviation of STAT1 domains: *ND* N-terminal domain, *CCD* coiled coil domain, *DBD* DNA-binding domain, *LD* linker domain, *SH2D* SH2 domain, *TD* transactivation domain

The full-length cDNA of STAT1 or STAT1-CC with Ala-656 to Cys-656 and Asn-658 to Cys-658 substitutions were amplified by PCR from plasmid Mx-STAT1-Flag-IRES-EGFP-neo and Mx-STAT1-CC-Flag-IRES-EGFP-neo that were generated as described previously [11]. The PCR products were then inserted into a lentiviral vector and confirmed by sequencing. The lentivirus particles were prepared in 293T cells as described previously [13, 14]. Lentiviral vectors encoding STAT1-IRES-EGFP and STAT1-CC-IRES-EGFP (Fig. 1) were transduced into lung cancer SPC-A-1 and H1299 cells to obtain stable STAT1- and STAT1-CC-expressing cells. Flow cytometry analysis of enhanced green fluorescent protein (EGFP) expression was used to detect the relative transduction efficiencies of the cell lines.

### Western blotting

Cells were lysed on ice with RIPA buffer (Sigma-Aldrich, St. Louis, MO, USA) containing protease inhibitor cocktail (Sigma-Aldrich). Protein content of the lysates was determined using the Bradford Protein Assay kit (Bio-Rad, Hercules, CA, USA). Equal amounts (40 μg/lane) of protein were separated by 12 % SDS PolyAcrylamide Gel Electrophoresis (SDS-PAGE) and transferred to polyvinylidene fluoride (PVDF) membranes (Millipore, Bedford, MA, USA). The blots were incubated with rabbit anti-PCNA antibody (Ab), rabbit anti-phospho-STAT1 (Tyr701) Ab (Cell Signaling Technology, Beverly, MA, USA), rabbit anti-S100A4 Ab (Abcam, Cambridge, UK), mouse anti-β-actin Ab (Santa Cruz Biotechnology, Santa Cruz, CA, USA), mouse anti-fibronectin Ab mouse anti-β-catenin Ab (Santa Cruz Biotechnology), mouse anti-STAT1 Ab, and mouse anti-c-fos Ab (Abcam). Primary Ab binding was detected with secondary antibody (anti-mouse or anti-rabbit HRP-conjugated IgG secondary antibody) that was detected using enhanced chemiluminescence substrate kit (GE Healthcare, Piscataway, NJ, USA). Quantification was performed with a ChemiDoc TM XRS + scanner and Image Lab Software (Bio Rad, CA, USA). The densities of each sample were normalized to the β-actin.

### Cell viability assay

Cells were seeded into 96-well plates at a density of 3 × 10^3^ cells/well. At the following time intervals (24, 48, 72, 96 and 120 h), cell viability was assayed using Cell Counting Kit-8 (CCK-8) assay kit (Dojin Laboratories, Kumamoto, Japan) according to the manufacturer’s instructions. To further examine whether STAT1-CC could better enhance IFN-induced growth inhibition in lung cancer cells, the cells transduced with STAT1, STAT1-CC, and EGFP were treated with IFN-γ or IFN-β and cell viability was measured using the CCK-8 assay. For IFN treatment, 1000 cells were seeded into 96-well plates and allowed to attach overnight. Cells were then treated with various concentrations of IFN-γ or IFN-β (R&D, Minneapolis, MN, USA) at different time intervals. Cell viability was assessed by CCK-8 assay kit.

### Cell apoptosis analysis

Cell apoptosis was examined by flow cytometry. Control as well as transduced lung cancer cells were plated into 6-well cell culture plates at a density of 6 × 10^4^ cells/well. After treatment with IFN-γ or IFN-β for 4 days, apoptosis was assessed by flow cytometry using the Annexin V apoptosis detection Kit APC from BD Bioscience (San Jose, CA, USA).

### Colony formation assay

Both control and transduced lung cancer cells were seeded into 10-cm culture dishes at a density of 5000 cells/dish and were grown in RPMI 1640 containing 10 % FBS for 14 days to form colonies. The colonies were then stained with Coomassie Blue and imaged accordingly.

### Cell migration and invasion assay

The effects of STAT1 and STAT1-CC on migration and invasive ability of lung cancer cells in vitro were examined using transwell assays. Transwell assays were performed in Costar transwell cell culture chamber inserts with an 8 μm pore size, placed in a 24-well cell culture plate (Corning Costar Corporation, Cambridge, MA, USA) [14]. Biological triplicates were performed for each of these assays.

For the cell migration assay, cells (1 × 10^5^) were suspended in serum-free medium and seeded to the upper part of chamber. Complete medium with 10 % FBS was added to the lower chamber as a chemoattractant. After 24 h, the transwell membrane was fixed with 4 % paraformaldehyde, and stained with 0.1 % cresyl violet. Migrated cells were counted under a microscope at 400× magnification. For the cell invasion assay, cells (1 × 10^5^) were suspended in serum-free medium and seeded into the upper compartment of the transwell chamber coated with 10 μl of diluted Matrigel (BD Biosciences). Complete medium with 10 % FBS was added to the lower chamber. After 48 h, cells were fixed, stained, and counted.

### Statistical analysis

Statistical significance was determined using the Student’s *t* test or analysis of variance (ANOVA). A P value of less than 0.05 is considered significant. Data are expressed as mean ± SEM.

## Results

### Expression of STAT1 or STAT1-CC significantly inhibits lung cancer cell growth

**Fig. 2:**
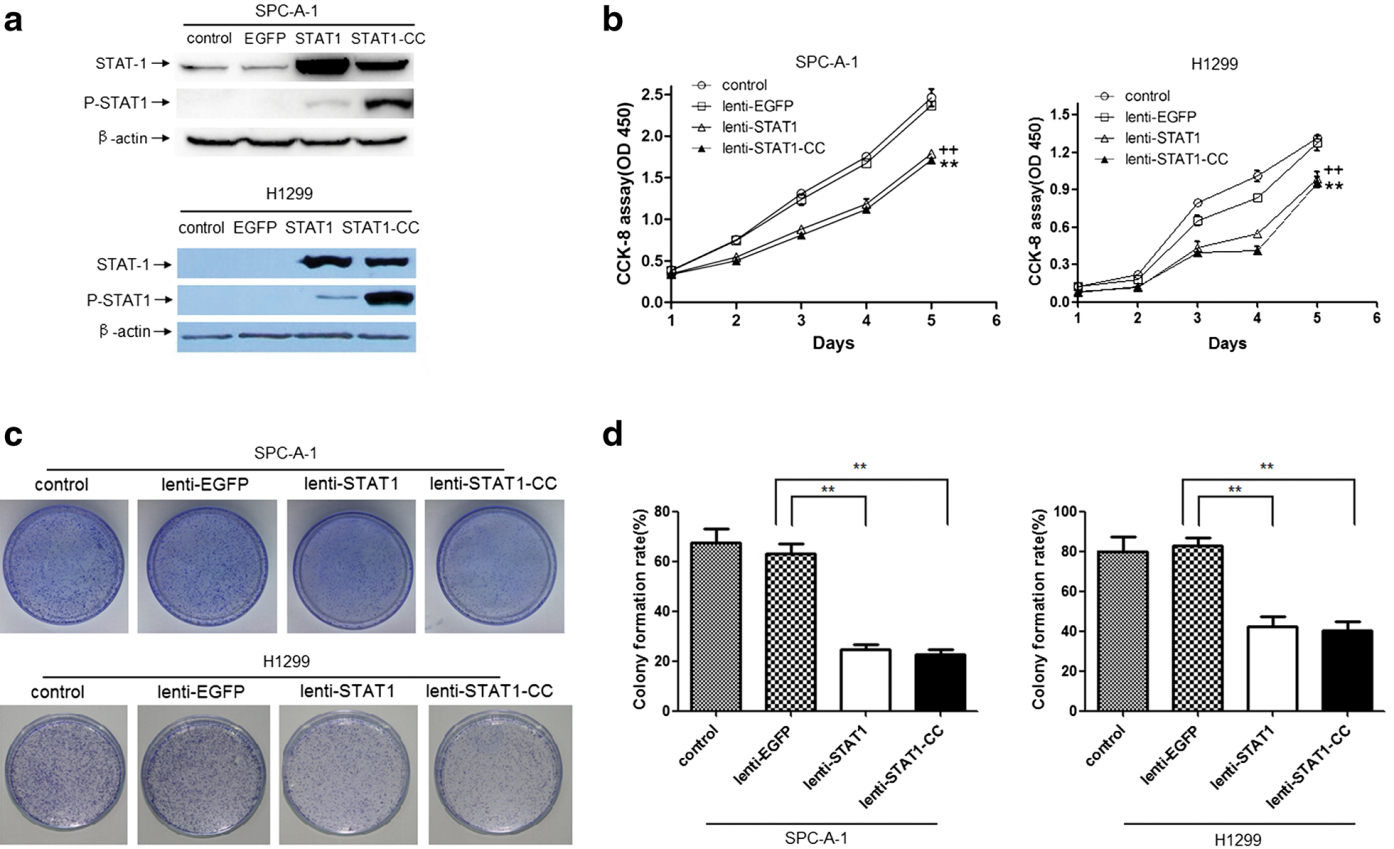
Overexpression of STAT1 or STAT1-CC induces growth inhibition of lung cancer cells. **a** Western blotting revealed whole cell levels of STAT1 and pSTAT1 in SPC-A-1 and H1299 cells transduced with lenti-STAT1 or lenti-STAT1-CC. **b** Overexpression of STAT1 or STAT1-CC resulted in significant reductions in the proliferation of SPC-A-1 and H1299 cells (^++^ *P* < 0.01, lenti-STAT1 vs lenti-EGFP; **P < 0.01, lenti-STAT1-CC vs lenti-EGFP). **c**, **d** Representative image of foci formation in a monolayer culture. The colony formation rates were significantly decreased in STAT1 and STAT1-CC-expressed SPC-A-1 and H1299 cells. Experiments were repeated at least three times. Data are shown as mean ± SEM. **P < 0.01

In order to overexpress wild type STAT1 and STAT1-CC mutant in lung cancer cells, we inserted these cDNA containing IRES-EGFP sequences into a lentiviral vector, and at the same time set up EGFP alone as a control using the same vector (Fig. 1). The transfection efficiencies (EGFP positive cells) of the stable EGFP-, STAT1- or STAT1-CC-expressing SPC-A-1 and H1299 cells were more than 95 % by flow cytometry analysis. Expression level of STAT1 or STAT1-CC was verified by western blot analysis. Interestingly, higher levels of pSTAT1 were detected in STAT1-CC transduced SPC-A-1 and H1299 cells at baseline compared to STAT1 transduced cells (Fig. 2a). Cell viability was determined using Cell Counting Kit-8 (CCK-8). As shown in Fig. 2b, STAT1 or STAT1-CC inhibited SPC-A-1 and H1299 lung cancer cell growth under basal culture conditions. Moreover, colony formation was significantly decreased in SPC-A-1 and H1299 cells transduced with STAT1 or STAT1-CC when compared to those in EGFP-expression cells. Colony formation rate for SPC-A-1 cells was 62.9 % in the EGFP controls and in cells transduced with STAT1 or STAT1-CC decreased to 24.7 and 22.6 % respectively. In H1299 cells, colony formation rate was reduced from 82.9 % in EGFP controls to 42.5 % with STAT1 and 40.1 % with STAT1CC cells (Fig. 2c, d). These data indicated that overexpression of STAT1 or STAT1-CC in lung cancer SPC-A-1 and H1299 cells inhibited cell proliferation and colony formation.

### Expression of STAT1 or STAT1-CC inhibits invasion and migration of lung cancer cells

**Fig. 3:**
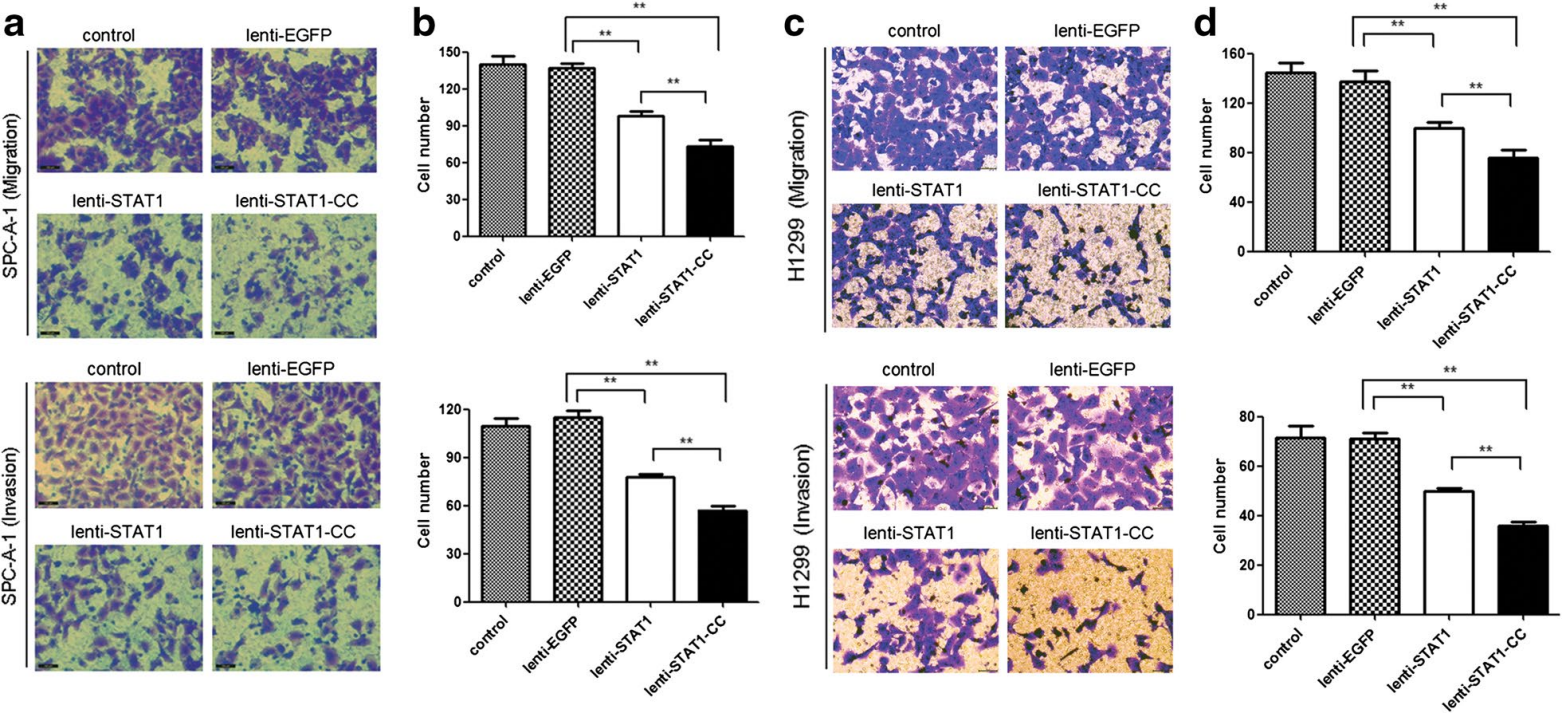
Overexpression of STAT1 or STAT1-CC inhibits the migration and invasion of lung cancer cells in vitro. **a, c** Representative images of migration and invasion from indicated groups (at ×400). Overexpression of STAT1 or STAT1-CC inhibited SPC-A-1 (**b**) and H1299 (**d**) cell migration and invasion. **P < 0.01

To examine the effect of STAT1 or STAT1-CC on the migration and invasiveness of lung cancer cells, we performed migration and invasion assays using transwell chambers. As shown in Fig. 3a, b, SPC-A-1 cells transduced with STAT1 or STAT1-CC displayed significantly decreased migration and invasive abilities when compared to either parent cells or cells transduced with EGFP. Reduced migration and invasiveness were also observed in H1299 cells transduced with STAT1 or STAT1-CC (Fig. 3c, d). STAT1-CC exhibited a stronger inhibitory effect on migration and invasiveness compared with wild type STAT1 in both lung cancer cell lines.

### STAT1-CC enhances IFNs-induced growth inhibition of lung cancer cells

**Fig. 4:**
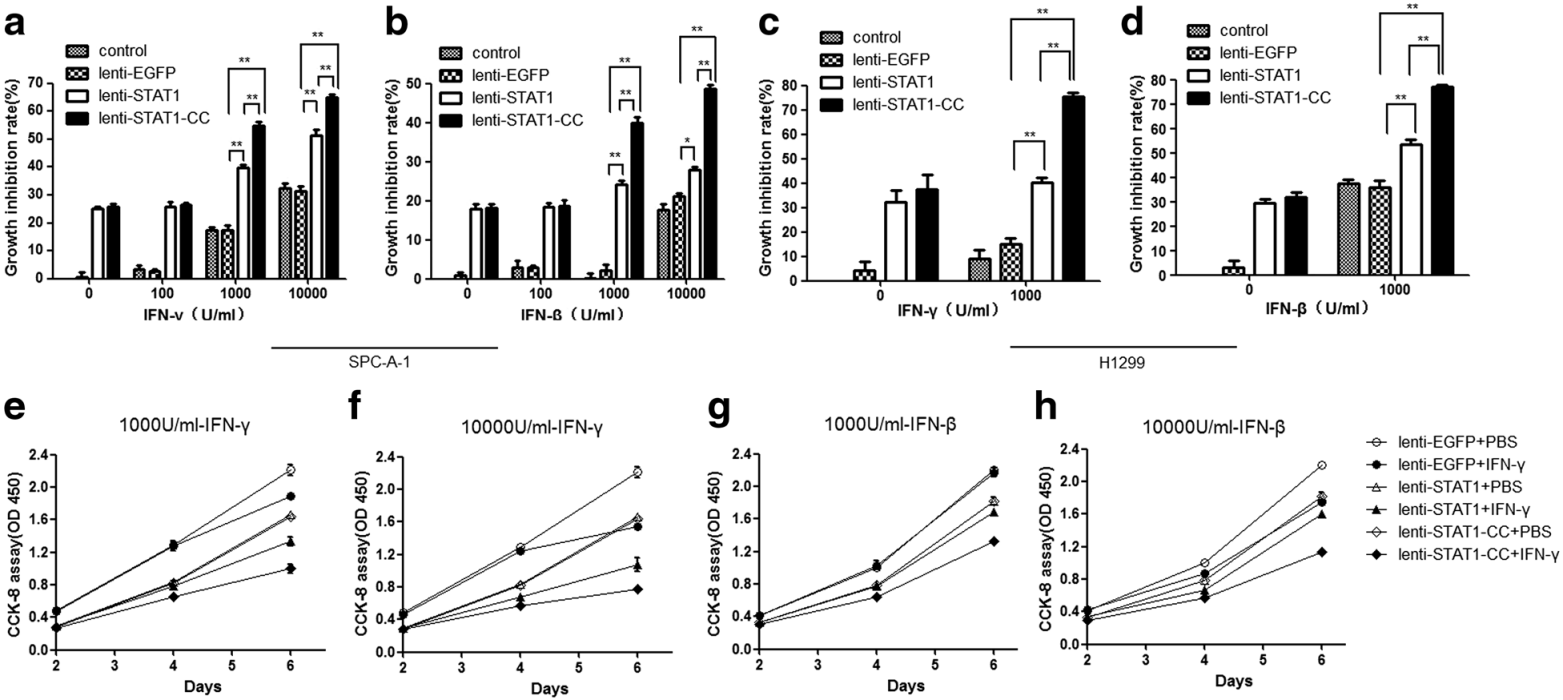
Overexpression of STAT1-CC improves IFN-induced growth inhibition in lung cancer cells. **a**, **b** SPC-A-1 cell growth inhibition rate was determined in each group treated with various doses of IFN-γ or IFN-β for 6 days (*P < 0.05, **P < 0.01). **c**, **d** H1299 cell growth was assayed in each group treated with IFN-γ 1000 U/ml or IFN-β 1000 U/ml for 2 days. **e**, **f** SPC-A-1 cell growth curve was plotted at various time points for each group treated with IFN-γ 1000 or 10,000 U/ml. **g**, **h** SPC-A-1 cell growth curves were plotted at various time points for each group treated with IFN-β 1000 or 10,000 U/ml

Under basal culture conditions, both STAT1 and STAT1-CC have similar effects on inhibition of lung cancer cell growth (Fig. 2b), but since in the previous studies, STAT1-CC demonstrated hyper-responsive to IFN stimulation [11], we stimulated the cells with various concentrations of IFNs. Although STAT1 effectively enhanced IFN-γ (1000 U/ml) or IFN-β (1000 U/ml) induced inhibition of growth at 39.7 and 24.1 %, respectively in SPC-A-1 cells, transduction with STAT1-CC resulted in a greater effect on inhibition of cell growth at 54.9 and 39.9 %, when cells were treated with the same concentrations of IFN-γ or IFN-β (Fig. 4a, b, e–h). A similar profile was observed in H1299-expressing STAT1 cells at inhibition rates of 40.2 and 53.5 % for IFN-γ and IFN-β, while inhibition rates of 75.4 and 77.0 % were achieved in STAT1-CC cells (Fig. 4c, d). These data indicated that STAT1-CC exhibits stronger inhibition of lung cancer cell growth with IFN treatment.

### STAT1-CC enhances IFNs-induced lung cancer cell apoptosis

**Fig. 5:**
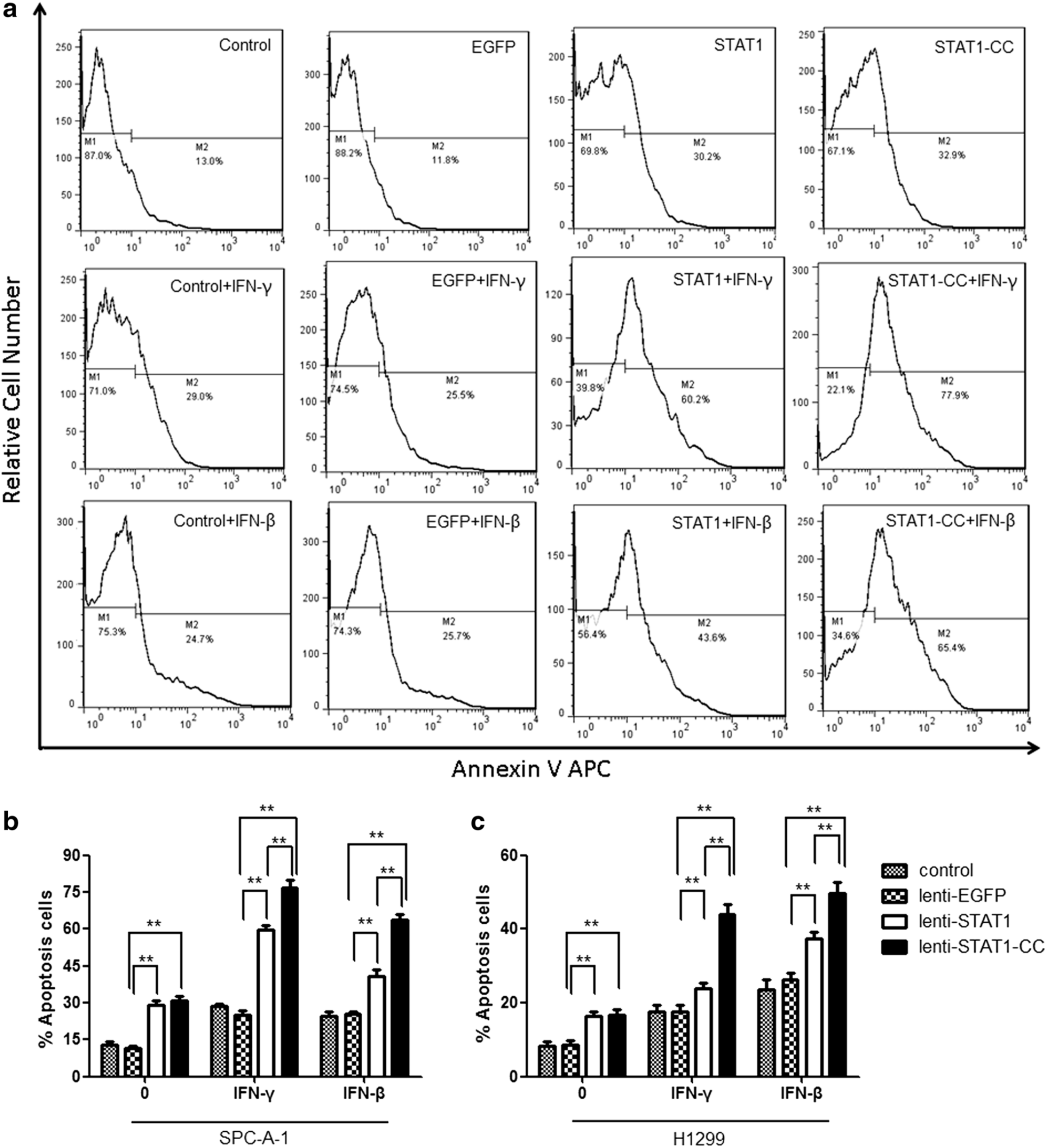
Overexpressing STAT1-CC enhances IFN-γ or IFN-β induced apoptosis of lung cancer cells. Apoptosis was detected by Annexin V apoptosis detection Kit and analysed by flow cytometry. **a** Flow cytometry results were demonstrated from control, EGFP-, STAT1-, and STAT1-CC-expressing SPC-A-1 cells treated with IFN-γ 10,000 U/ml or IFN-β 10,000 U/ml for 96 h. **b** Quantitative analysis of the population of SPC-A-1 apoptotic cells. **c** Control, EGFP-, STAT1-, and STAT1-CC-expressing H1299 cells were treated with IFN-γ 1000 U/ml or IFN-β 1000 U/ml for 48 h. Quantitative analysis of the population of apoptotic cells in Three independent experiments were conducted and data are shown as mean ± SEM. **P < 0.01

IFNs have potent antiproliferative and apoptosis inducing functions in several types of cancer cells. To examine whether overexpression of STAT1 or STAT1-CC will affect apoptotic events in the transduced lung cancer cells, apoptosis was measured using Annexin V apoptosis assay. STAT1 or STAT1-CC induced apoptosis in transduced SPC-A-1 cells under normal culture conditions. More importantly, STAT1- or STAT1-CC-expressing lung cancer SPC-A-1 cells showed increased levels of apoptosis compared to parent and EGFP expressing cells when treated with either IFN-γ (10,000 U/ml) or IFN-β (10,000 U/ml) for 96 h (Fig. 5). Notably, when treated with IFN-γ or IFN-β, SPC-A-1 cells transduced with STAT1-CC exhibited a significantly increased rate of apoptosis compared to cells transduced with wild type STAT1.

### STAT1-CC tyrosine phosphorylation was increased in transduced lung cancer cells

**Fig. 6:**
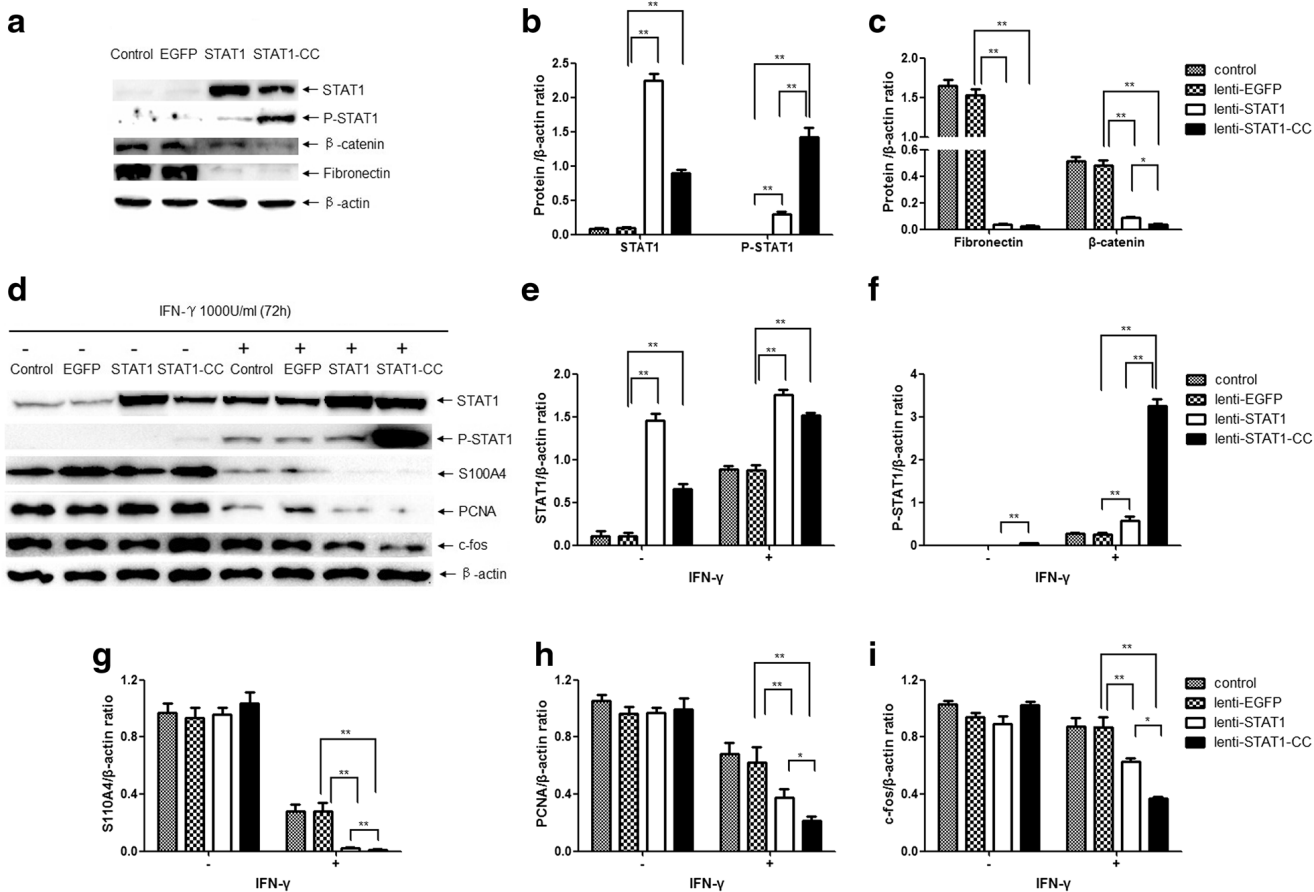
STAT1-CC increases STAT1 phosphorylation in lung cancer cells. **a** Western blot for pSTAT1, fibronectin and β-catenin in STAT1- or STAT1-CC-expressing SPC-A-1 cells. **d** Western blot for pSTAT1, S100A4, PCNA and c-fos in STAT1- or STAT1-CC-expressing SPC-A-1 cells treated with IFN-γ (1000 U/ml) for 72 h. β-actin was used as a loading control. **b**, **c**, **e**–**i** Western blot levels of indicated proteins were quantified with the ChemiDoc ™ XRS+ scanner and Image Lab Software imaging system in the SPC-A-1 cells. The densities of each sample were normalized to the β-actin

STAT1 Tyr-701 phosphorylation is an essential step for STAT1 activation. As shown in Fig. 6a, overexpression of STAT1 or STAT1-CC in SPC-A-1 cells promoted STAT1 tyrosine phosphorylation under basal conditions. STAT1-CC-expressing cells have a higher pSTAT1 level, although the protein level is 2.2- to 2.5-fold lower than that in STAT1-expressing cells. To better understand the decrease in migration and invasiveness of STAT1-CC cells, we examined several factors involved in cell growth, inhibition, migration and invasion. Expression of fibronectin was reduced at similar levels in STAT1- and STAT1-CC-expressing cells, while there was a greater decrease of β-catenin expression in STAT1-CC cells compared to STAT1-expressing cells (Fig. 6a, b). In the case of IFN-γ stimulation, the level of pSTAT1 in STAT1-CC-expressing cells is increased 12.6-fold whereas the pSTAT1 level in STAT1-expressing cells is only increased 2.2-fold compared to that of EGFP-expressing cells (Fig. 6d, f). Moreover, overexpression of STAT1 or STAT1-CC reduced S100A4, PCNA, and c-fos expressions when treated with IFN-γ, greater decreased levels of these proteins were observed in STAT1-CC cells compared to STAT1-expressing cells (Fig. 6d). The quantified data are shown in Fig. 6b, c and e–i.

## Discussion

IFN-γ receptor (*ifngr1*)-deficient or *Stat1*-deficient mice developed tumors more rapidly compared to wild-type mice for both chemically induced and spontaneously arising tumors [15]. These data demonstrate that STAT1 may function as a tumor suppressor. Some spontaneous human tumors have been reported to be selectively unresponsive to IFN-γ due to impaired STAT1 activation and this suggests that as in mouse models, the IFN-STAT1 dependent tumor surveillance mechanism also engages in humans [16]. Type I and Type II IFNs have been used in the treatment of many types of cancers [4]. However, IFNs have limited clinical utility in solid tumors, largely due to their half-life which makes it difficult to achieve sustained therapeutic concentrations [3, 5]. In order to overcome these limitations, many investigators have attempted to improve the effects of IFNs through modification of IFN signaling [11]. Inhibition of endogenous inhibitors of STAT1 phosphorylation (e.g. SOCS1) or DNA binding (e.g. PIAS1) could be used to modify IFN signaling [11, 17, 18]. STAT1-CC with double-cysteine substitutions in the Src homology 2 (SH2)-homodimerization domain markedly increases responsiveness to both types I and II IFNs [11]. The mechanism for improving IFN efficacy depends on increased capacity for transcriptosome assembly at the promoter of interferon-stimulated genes (ISGs) that is manifested by prolonged STAT1 Tyr-701 phosphorylation, DNA-binding and p300/CBP co-activator recruitment [11]. STAT1-CC was also shown to be able to overcome IFN-γ resistance and induce a hepatitis C virus antiviral response in IFN-α resistant replicon cells [12].

In this study, STAT1 and STAT1-CC genes were successfully overexpressed in lung cancer cells using the lentivirus-mediated gene transfer system. STAT1 is considered to play a significant role in regulating cell proliferation and apoptosis [19]. Previous studies have shown that STAT1 controls anti-tumorigenic functions by upregulating the expressions of caspases 1, 2, 3, 7, and 8 [20–23] or inducible nitric oxide synthase (iNOS) [19], p21 [24] and IRF1/p53 pathway [25], and downregulating expressions of c-myc [26], and the BCL2 family [27]. STAT1 also acts as a negative regulator of tumor angiogenesis partially through the induction of CXCL10 [28]. Here, we have examined several factors which have been reported to be involved in cell growth, migration, invasion and apoptosis. Fibronectin has been recognized as an important protein for cancer invasiveness and metastasis [29, 30], and β-catenin has been shown to play an essential role in lung tumorigenesis [31]. The levels of both proteins were decreased in STAT1 and STAT1-CC transduced lung cancer cells, but there was a greater reduction of β-catenin in STAT1-CC cells. STAT1 undergoes multiple types of post-translational modifications, e.g. acetylation and ubiquitination in addition to phosphorylation. Although we have found that more p300/CBP co-activator is recruited to the ISG transcriptional complex in STAT1-CC transduced fibrosarcoma cells [11], the acetylation level of STAT1-CC remains unclear. Kramer et al., have shown that acetylated Stat1 is able to interact with NF-κB p65, resulting in decreased p65 DNA binding, nuclear localization, and expression of anti-apoptotic NF-κB target genes [32]. Lee et al., reported that NF-κB activates fibronectin gene expression in rat hepatocytes [33]. It will be interesting to look at the acetylation level of STAT1-CC in lung cancer cells. β-catenin has been reported to physically interact with NF-κB to form a complex, resulting in reduced NF-κB DNA binding and target gene expression. Moreover, a strong inverse correlation was found between the expression levels of β-catenin and Fas in colon and breast tumor tissues [34]. We will therefore examine whether Fas level was increased in STAT1-CC cells. S100A4 is associated with increased cell growth and metastatic capacity of lung cancer cells, and, PCNA plays an important role in DNA replication and repair. It has been revealed that PCNA is a potential anticancer target [35]. Both S100A and PCNA expression are suppressed by IFN-γ [36, 37]. c-fos proto-oncogene is described as an immediate early response gene, in which the high expression of c-fos can induce the formation of tumors [38]. Interferon-α-2b downregulates the expression of c-fos, H-ras, c-raf-2, c-kit, c-myc, and c-myb in a hairy cell leukemic line [39]. Expression of S100A4, PCNA and c-fos were reduced in STAT1- and STAT1-CC-expressing lung cancer cells, while PCNA and c-fos were decreased to a greater extent in STAT1-CC cells. STAT1 phosphorylation is higher in STAT1-CC cells both at baseline and with IFN-γ treatment. This indicates that the IFN-STAT1 signaling pathway is more active in this cell line, and might represent a potential molecular mechanism in which STAT1-CC could enhance the antitumor response of IFNs through upregulation of gene expression involved in inhibition of cell growth and metastasis in lung cancer cells. The mechanism for regulation of these genes needs further study.

The delivery of therapeutic genes into lung cancer cells is still a challenging approach in gene therapy. Recently, Powell et al., reviewed methods by which viral vectors can be engineered to enhance target specificity and increase transgene expression [40]. On the other hand, the development of small molecules that are able to enhance IFN signaling would validate a roadmap for next generation drugs for treatment of interferon-sensitive tumors [41, 42].

## Conclusions

In conclusion, overexpression of STAT1 or STAT1-CC inhibits human lung cancer cell proliferation, migration and invasion, and enhances the antitumor response of IFNs in vitro. STAT1-CC has stronger IFN induced antitumor activity than STAT1 through enhanced STAT1 phosphorylation in lung cancer cells. Together, these data suggest that combined treatment of IFNs and STAT1-CC may represent a useful therapeutic strategy for the clinical management of human lung cancer, though this approach must be evaluated for the safety and efficacy by clinical trials before any clinical applications.

## Abbreviations

IFNs: interferons
STAT1-CC: Ala-656 and Asn-658 sites with double-cysteine alternative in the SH2 domain of STAT1
IFN-γ: interferon-gamma
IFN-β: interferon-beta
JAK-STAT: the Janus activated kinase-signal transducer and activator of transcription
pSTAT1: STAT1 Tyr-701 phosphorylation
FBS: fetal bovine serum
CCK-8: cell counting kit-8
SDS-PAGE: SDS polyacrylamide gel electrophoresis
Ab: antibody
PVDF: polyvinylidene fluoride
PCNA: proliferating cell nuclear antigen
ANOVA: analysis of variance
EGFP: enhanced green fluorescent protein
iNOS: inducible nitric oxide synthase
ISGs: interferon-stimulated genes
